# Development of prognostic index based on autophagy-related genes analysis in breast cancer

**DOI:** 10.18632/aging.102687

**Published:** 2020-01-22

**Authors:** Qing-Guang Lin, Wei Liu, Yu-zhen Mo, Jing Han, Zhi-Xing Guo, Wei Zheng, Jian-wei Wang, Xue-Bin Zou, An-Hua Li, Feng Han

**Affiliations:** 1 Department of Ultrasound, State Key Laboratory of Oncology in South China, Collaborative Innovation Center for Cancer Medicine, Sun Yat-Sen University Cancer Center, Guangzhou 510060, Guangdong, China; 2 Department of Breast, Guangzhou Red Cross Hospital, Medical College, Jinan University, Guangzhou 510220, Guangdong, China; 3 Department of Radiotherapy, Guangzhou Red Cross Hospital, Medical College, Jinan University, Guangzhou 510220, Guangdong, China

**Keywords:** autophagy-related genes, breast cancer, prognosis

## Abstract

Background: Autophagy is a self-digesting process that can satisfy the metabolic needs of cells, and is closely related to development of cancer. However, the effect of autophagy-related genes (ARGs) on the prognosis of breast cancer remains unclear.

Results: We first found that 27 ARGs were significantly associated with overall survival in breast cancer. The prognosis-related ARGs signature established using the Cox regression model consists of 12 ARGs that can be divided patients into high-risk and low-risk groups. The overall survival of patients with high-risk scores (HR 3.652, 2.410-5.533; P < 0.001) was shorter than patients with low-risk scores. The area under the receiver operating characteristic (ROC) curve for 1-year, 3-year, and 5-year survival rates were 0.739, 0.727, and 0.742, respectively.

Conclusion: The12-ARGs marker can predict the prognosis of breast cancer and thus help individualized treatment of patients at different risks.

Methods: Based on the TCGA dataset, we integrated the expression profiles of ARGs in 1,039 breast cancer patients. Differentially expressed ARGs and survival-related ARGs were evaluated by computational difference algorithm and COX regression analysis. In addition, we also explored the mutations in these ARGs. A new prognostic indicator based on ARGs was developed using multivariate COX analysis.

## INTRODUCTION

Autophagy is a multi-step lysosomal degradation process that promotes nutrient cycling and metabolic adaptation, and has been extensively studied and been proven involved in the development of cancer [1]. However, the function of autophagy in tumors is bilateral, may be cancer-promoting, or may be a tumor suppressor, depending on the type of tumor and the stage of the tumor [2]. For example, autophagy can remove damaged organelles and/or DNA before canceration to maintain normal cellular structure and metabolic stability, thereby exerting a tumor suppressing effect [3]. To the stage of tumor progression, autophagy is often up-regulated and promotes tumor cell proliferation and invasion by absorbing nutrients and energy from degrading proteins and organelles [4]. Autophagy is a complex multi-step process that is tightly controlled by a series of autophagy-related genes (ARGs).

The incidence of breast cancer ranks first among women in cancer worldwide, and it is a significant threat to the health of women [5, 6]. It is well known that breast cancer is a group of highly heterogeneous diseases, and the prognosis of individuals varies widely [7]. Clinically, tumor staging, histological grades, and molecular subtypes are used to evaluate the prognostic factors of breast cancer patients. However, these clinicopathological features do not accurately provide information to predict a patient's prognosis. This may lead to inaccurate judgments on the patient's prognosis, and some low-risk patients may receive unnecessary or excessive treatment, while other high-risk patients may face relapse or metastasis due to inadequate treatment. Therefore, there is an urgent need to find new molecular markers to predict the prognosis of breast cancer patients, which is conducive to the precise treatment of patients.

A large number of studies have reported a correlation between autophagy and breast cancer [8–10]. For example, Vera-Ramirez et al. reported that autophagy promotes the therapeutic resistance of breast cancer stem cells and contributes to its survival [11]. Notable, previous studies have focused on the association between single or a few ARGs and breast cancer progression. Currently, studies using large-scale ARGs expression profiles to screen and identify molecular markers for predicting the prognosis of breast cancer are lacking. The purpose of this study was to gain insight into the potential clinical utility of ARGs for prognostic stratification and to facilitate the development of personalized prognostic information for breast cancer patients. We combined ARGS expression profiles with clinical information to systematically analyze the expression status of ARGS and its impact on prognosis.

## RESULTS

### Identification of differentially expressed ARGs

**Figure 1:**
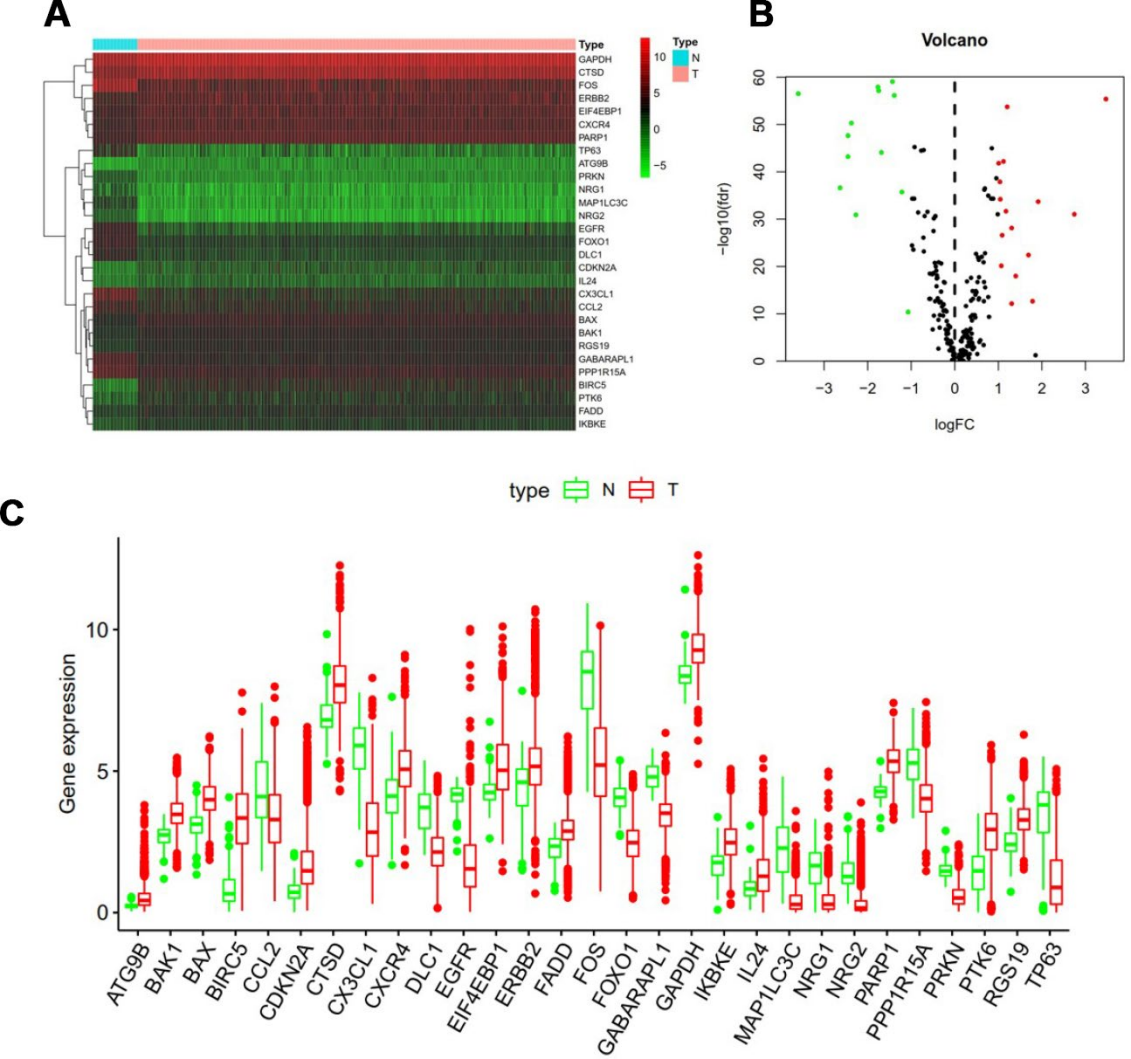
**Differentially expressed autophagy-related genes.** Heat map (**A**) and volcano map (**B**) show differentially expressed genes between breast cancer and normal tissues, with red dots representing significantly up-regulated genes, green dots representing significantly down-regulated genes, and black dots representing no differences gene. (**C**) Expression patterns of 29 autophagy-related genes (ARGs) in breast cancer types and paired non-tumor samples. Each red box plot represents a different tumor sample and blue represents a non-tumor sample.

RNA-seq and clinical data from 1109 breast cancer tissue samples and 113 non-tumor samples were downloaded from TCGA. Of these patients, a total of 1039 patients with primary breast cancer who were followed for more than 1 month were included in the study. The expression values of 232 ARGs were extracted. Considering the criteria for FDR <0.05 and [log2 (fold change)]> 1, we finally obtained 13 up-regulated and 16 down-regulated ARGs (Figure 1A and 1B). A detailed flow chart for the establishment of the prediction model was shown in Supplementary Figure 1.

In addition, a scatter plot was visualized to show the expression pattern of 29 differentially expressed ARGs between breast cancer and non-tumor tissue (Figure 1C). Scatter plot showing expression patterns of 13 down-regulated genes (*CCL2, DLC1, EGFR1, CX3CL1, FOS, FOXO1, GABARAPL1, MAP1LC3C, NRG1, NRG2, PPP1R15A, PRKN* and *TP63*) and 16 Up-regulated genes (*ATG9B, BAK1, BAX, BIRC5, CDKN2A, CTSD, CXCR4, EIF4EBP1, ERBB2, FADD, GAPDH, IKBKE, IL24, PARP1, PTK6* and *RGS19*).

### Functional enrichment of the differentially expressed ARGs

**Figure 2:**
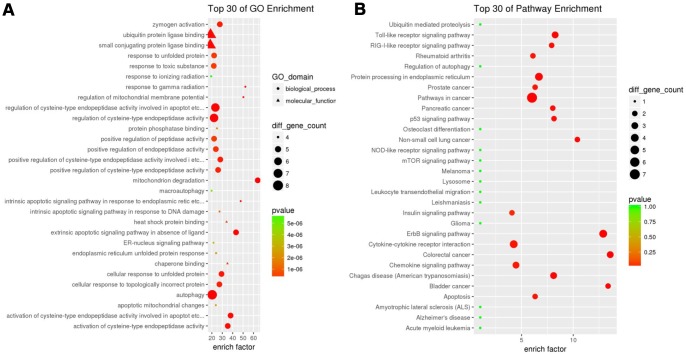
**Gene functional enrichment of differentially expressed ARGs.** (**A**) GO analysis shows the biological processes and molecular functions involved in differential genes. (**B**) KEGG shows the signaling pathway involved in differential ARGs.

Functional enrichment analysis of 29 differentially expressed ARGs provides a biological understanding of these genes. Top 30 of GO enrichment and top 30 of pathway enrichment are summarized in Figure 2A. GO enrichment shows that the biological process of differential genes is mainly involved in autophagy, apoptosis and endopeptidase regulation. KEGG enrichment shows that pathways of differential genes mainly involve pathways in cancer, protein processing in endoplasmic reticulum, cytokine-cytokine receptor interaction and the like (Figure 2B).

### Identification of prognostic ARGs

**Figure 3:**
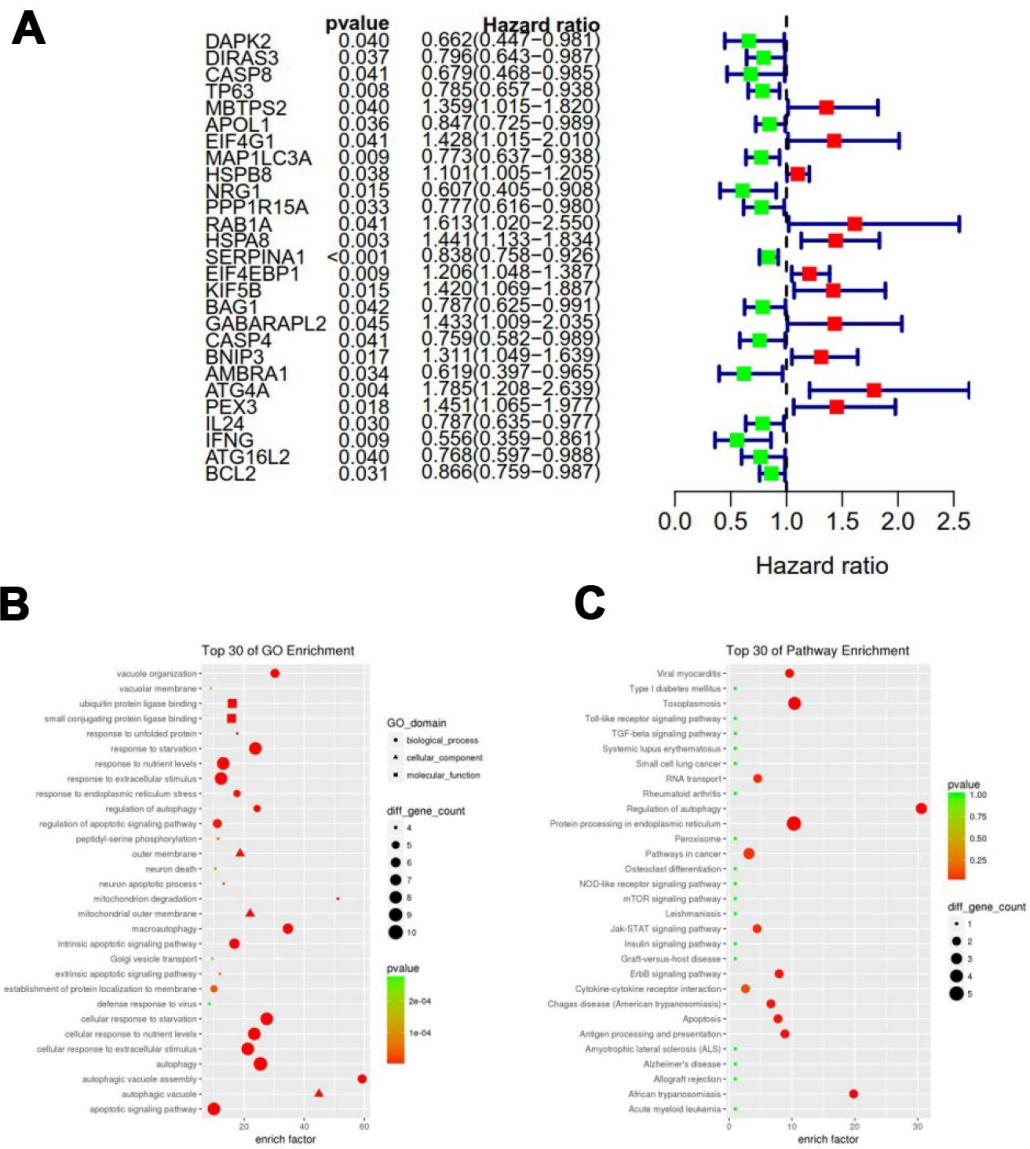
**Expression profile and prognostic value of ARGs.** (**A**) Risk ratio forest plot showed the prognostic value of the gene; (**B**) GO analysis revealed the biological processes and molecular functions involved in 27 prognostic-related ARGs; (**C**) KEGG shows the signaling pathways involved in 27 prognostic-related ARGs.

**Figure 4:**
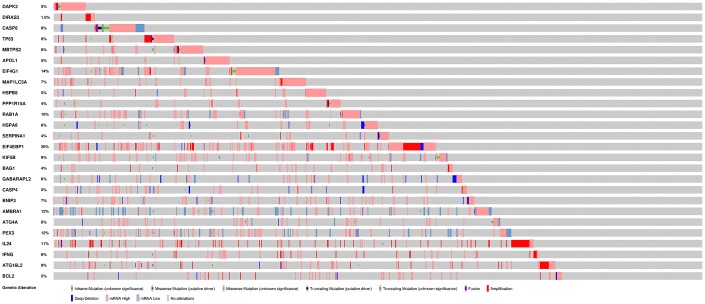
**Mutations in prognosis-related ARGs.** *EIF4EBP1* is the most frequently mutated gene. A total of 22 genes have a mutation rate ≥ 5%.

To analyze ARGs involved in breast cancer progression, we screened for ARGs that were significantly associated with prognosis. The forest map of the hazard ratio indicates that most of these genes are protective factors (Figure 3A). Both GO and KEGG analysis showed that these genes are closely related to autophagy-related biological processes and signaling pathways (Figure 3B and 3C). Given the important clinical implications of these ARGs, we examined the genetic alterations of these genes and found that mRNA up-regulation and fusion are the two most common types of mutations (Figure 4). A total of 22 genes have a mutation rate ≥ 5%, of which *EIF4EBP1* is the most frequently mutated gene (20%).

Prognostic ARGs with significant significance after the above univariate analysis were further included in the subsequent multivariate analysis. A total of 12 genes were significantly associated with prognosis after multivariate analysis. The expression patterns of these 12 genes were shown in supplementary Figure 2. Protein-protein interaction (PPI) network analysis indicated that *EIF4G1, CASP8* and *MAP1C3CA* are the three core genes of these 12 genes (Supplementary Figure 3). Based on the results of multivariate Cox regression analysis, we constructed autophagy prognostic index (API) to divide breast cancer patients into two groups with discrete clinical outcomes for overall survival (OS). [Expression level of CASP8* (-0.5681)] + [Expression level of *EIF4G1* * (0.3535)] + [Expression level of *MAP1LC3A* * (-0.2183)] + [Expression level of *HSPB8* * 0.1302] + [Expression level of *NRG1* * (-0.6381)] + [Expression level of SERPINA1* (-0.1772) + [Expression level of *EIF4EBP1* * (0.1618)]+ [Expression level of BAG1* (-0.2884)] + [Expression level of *CASP4* * (0.3839)] + [Expression level of *AMBRA1* * (-0.4073)] + [Expression level of *ATG4A* * 0.6891] + [Expression level of *IFNG* * (-0.9043)].

**Figure 5:**
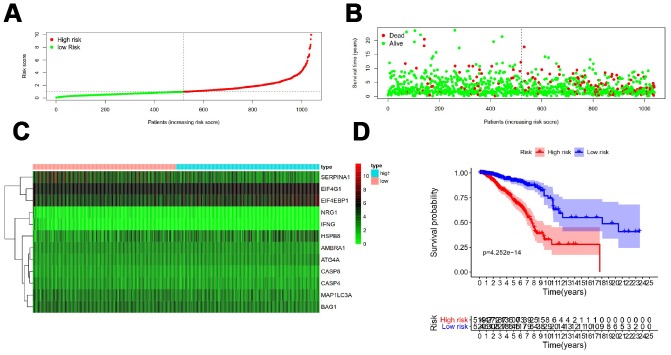
**Development of a prognostic index based on ARGs.** (**A**) Distribution of prognostic index. (**B**) Survival status of patients in different groups. (**C**) Heat map of the expression profile of the included ARGs. (**D**) Patients in the high-risk group have a shorter overall survival.

**Figure 6:**
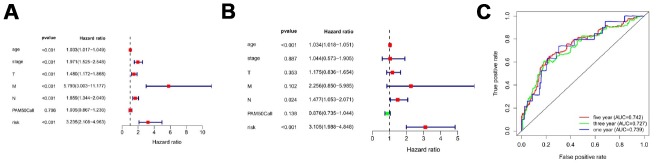
**Prognostic indicators based on ARGs show good predictive performance.** A forest plot of univariate (**A**) and multivariate (**B**) Cox regression analysis in breast cancer. (**C**) Survival-dependent receiver operating characteristic (ROC) curves validate the prognostic significance of ARGs-based prognostic indicators.

Figure 5 showed distribution of prognostic index in TCGA dataset (Figure 5A), survival status of patients in different groups (Figure 5B) and heatmap of the expression profile of the included ARGs (Figure 5C). To determine the performance of the API in predicting clinical outcomes in breast cancer patients, K-M survival curves were plotted to analyze different survival times between high-risk and low-risk groups. K-M analysis showed that the survival rate of patients in the high-risk group was significantly lower than that in the low-risk group (Figure 5D). Univariate analysis showed that ARI was significantly associated with patient prognosis (Figure 6A). In addition, after adjusting for clinicopathological features such as age, tumor subtype, tumor stage, tumor size, and lymph node metastasis, API remained an independent prognostic indicator for breast cancer patients in multivariate analysis (HR = 3.105, 95% CI = 1.988-4.848; *P* < 0.001; Figure 6B). The area under the curve of the corresponding receiver operating characteristic (ROC) curve for 1 year, 3 years, and 5 years of survival is 0.739, 0.727, and 0.742, respectively. This indicated that the prognostic index based on ARGs has a certain potential in survival prediction (Figure 6C).

### Clinical utility of prognostic signature

**Figure 7:**
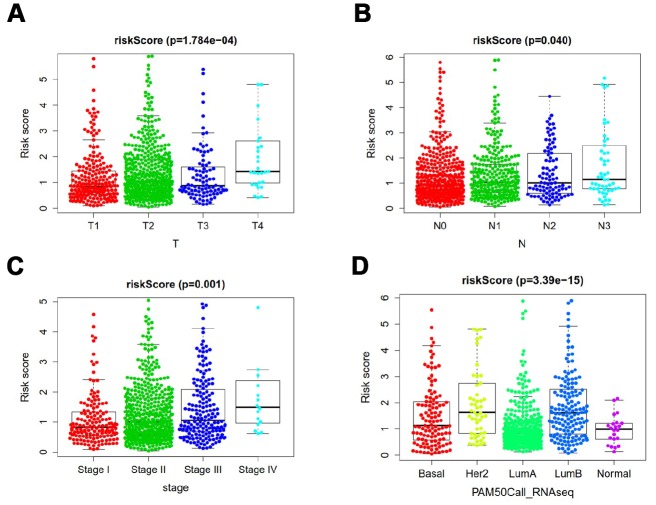
**Clinicopathological significance of the prognostic index of breast cancer.** P values were at different (**A**) tumor size, (**B**) lymph node metastasis (**C**) tumor stage, and (**D**) tumor subtypes.

Relationship between ARGs prognostic index and clinical features were subsequently analyzed. Significant increases in risk score were in larger tumor size (Figure 7A), lymph node metastasis (Figure 7B), late clinical stage (Figure 7C), HER2 subtype and luminal B subtype (Figure 7D).

## DISCUSSION

Although numerous studies have demonstrated that autophagy is involved in the malignant progression of breast cancer, a comprehensive analysis of ARGs have not been conducted to explore its clinical significance. To analyze breast cancer prognosis-related genes from the perspective of autophagy, we screened and identified 12 prognostic ARGs. Our results suggested that a prognostic model based on 12 ARGs can be used for prognostic stratification in breast cancer patients, thereby helping to develop individualized treatment options based on patient risk.

We identified a group of ARGs that predict the prognosis of breast cancer patients. Most of these genes have been reported in previous studies to be closely related to the prognosis of breast cancer or other malignancies [12]. Loss of CASP8 protein expression is associated with a poor prognosis in children with medulloblastoma [13]. Muhammad JS et al. [11] reported that *Helicobacter pylori*-induced *MAP1LC3* methylation silencing may impair autophagy and promote gastric cancer. Overexpression of EIF4G1 is associated with tumor progression and poor prognosis in nasopharyngeal carcinoma [14]. HSPB8 promotes cancer cell growth and is associated with poor prognosis in patients with gastric cancer [15]. Overexpression of phosphorylated EIF4EBP1 is closely associated with tumor recurrence and worse survival outcomes of cervical cancer [16]. Afentakis M et al reported that the incidence of distant recurrence in women with higher BAG1 expression was reduced by 30% compared with women with low expression of breast cancer [17]. The circulating NRG1 reported by De Iuliis F et al. may be a biomarker for the prognosis of breast cancer patients [18]. Boccellino M et al found that SERPINA1 may be a useful biomarker for early detection of lung cancer and monitoring its evolution [19]. ATG4A has been reported to promote tumor metastasis by inducing epithelial-mesenchymal transition and stem cell-like properties of gastric cells [20]. In triple-negative breast cancer, high expression of IFNG was found to be associated with better disease-free survival. The focus of this study was on the relationship between the mRNA expression of ARGs and the prognosis of breast cancer patients. It is known that the genetic alternation of genes is likely to affect the expression levels of their mRNAs. Gene amplification is often positively correlated with up-regulation of mRNA expression. For example, we noted that gene amplification and mRNA upregulation are the most common genetic variants for the *EIF4EBP1*.

There are still some limitations in this study. First, our research is a retrospective study, so there may be some inherent bias. Second, the prognostic model still needs to be further validated in other independent cohorts to ensure the robustness of our established model. Third, functional experiments are needed in the future to further reveal the potential mechanisms for predicting the role of autophagy genes.

In conclusion, this study identified multiple breast cancer prognostic ARGs based on a comprehensive analysis of ARGs expression profiles and corresponding clinical features. The genes identified in the autophagy pathway also offer new possibilities for breast cancer therapeutic intervention. Based on the molecular features of autophagy, we constructed a new risk scoring model that can effectively assess the prognosis of breast cancer patients. However, prospective studies are needed to further validate the findings of this study to aid clinical personalized treatment.

## MATERIALS AND METHODS

### Autophagy related genes (ARGs)

A total of 232 ARGs were extracted from the HADB database (Human Autophagy Database, http://autophagy.lu/clustering/index.html), which provides a complete, up-to-date list of human genes involved in autophagy.

### TCGA data acquisition

Our study included only 1039 breast cancer patients who were followed up for at least one month from the TCGA database, with a follow-up time ranging 1to 283 months. ARGs associated with patient survival were identified using univariate Cox regression for subsequent model construction.

### Functional analysis

The Bohao Online Enrichment Tool (http://enrich.shbio.com/) was used to perform functional enrichment of differentially expressed ARGs. Gene Ontology (GO) and the Kyoto Gene and Genomic Encyclopedia (KEGG) were used to assess relevant functional categories. GO and KEGG enrichment pathways with p and q values less than 0.05 are considered to be significant categories.

### Construction of ARGs related prognostic model

Prognosis-related genes were constructed using multivariate cox regression. After incorporating the expression values for each particular gene, a risk score formula for each patient was constructed and weighted by its estimated regression coefficients in a multivariate cox regression analysis. According to the risk scoring formula, the median risk score was used as the cut-off point, and the patients were divided into low-risk group and high-risk group. Survival differences between the two groups were assessed by Kaplan-Meier and compared using log-rank statistical methods. Multivariate cox regression analysis and stratified analysis were used to examine the role of risk scores in predicting patient outcomes. ROC curves were used to study the accuracy of model predictions.

### Statistical analysis

Survival curves were generated by the Kaplan-Meier method and compared by log-rank test. Multivariate analysis was performed using the cox proportional hazard model. All statistical analyses were performed using the R language (version 3.6). All statistical tests were bilateral, with p < 0.05 being statistically significant.

## Supplementary Material

Supplementary Figures
