# Supplementary material for: Development of prognostic index based on autophagy-related genes analysis in breast cancer
Source: Aging (Albany NY). 2020 Jan 22;12(2):1366–76. doi: 10.18632/aging.102687 (PMC7053636; doi:10.18632/aging.102687)
Supplement: Supplementary Figures [file aging-12-102687-s001..pdf]

SUPPLEMENTARY FIGURES

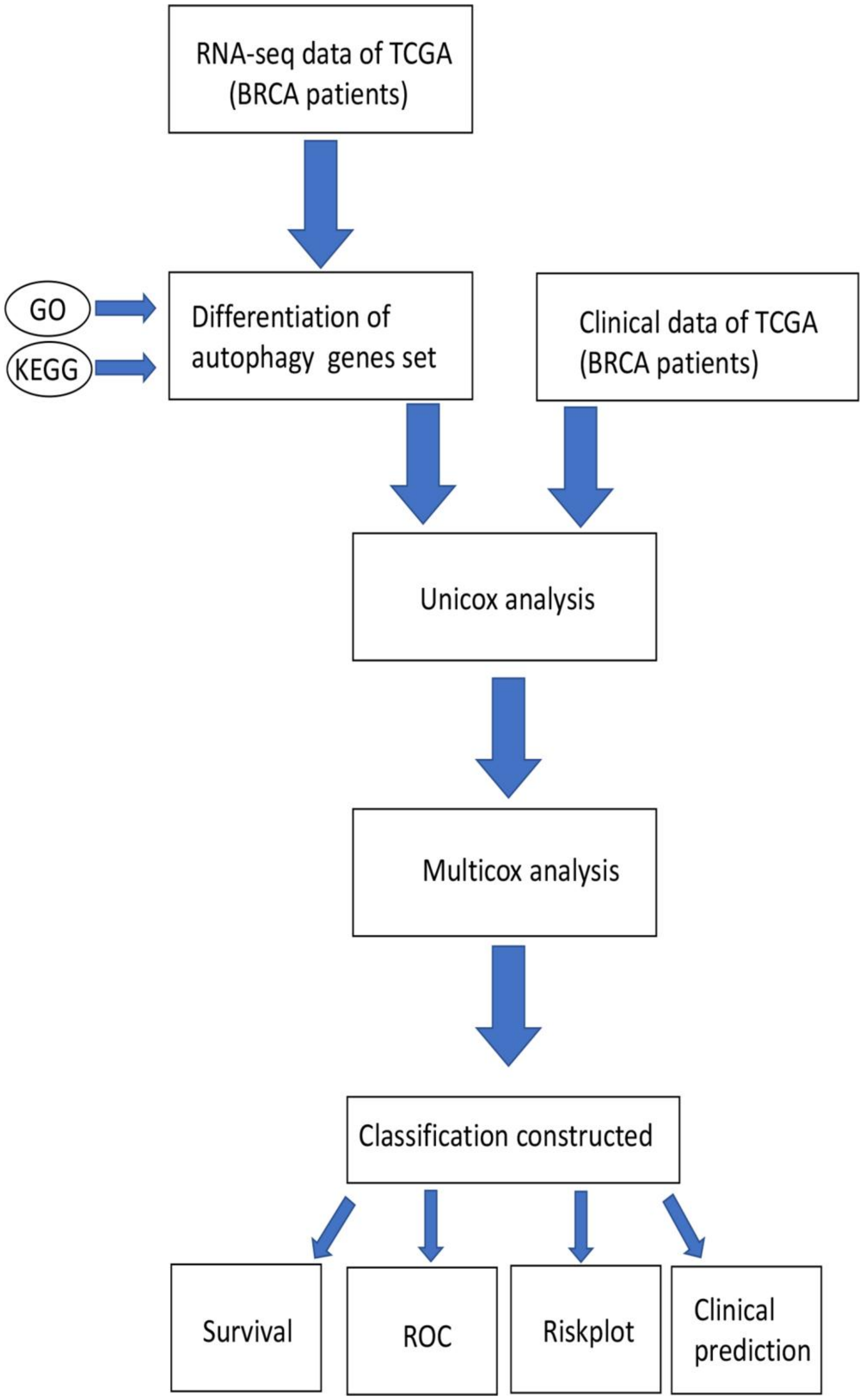

Supplementary Figure 1. Flowchart for identifying 12 ARGs signature associated with breast cancer survival.

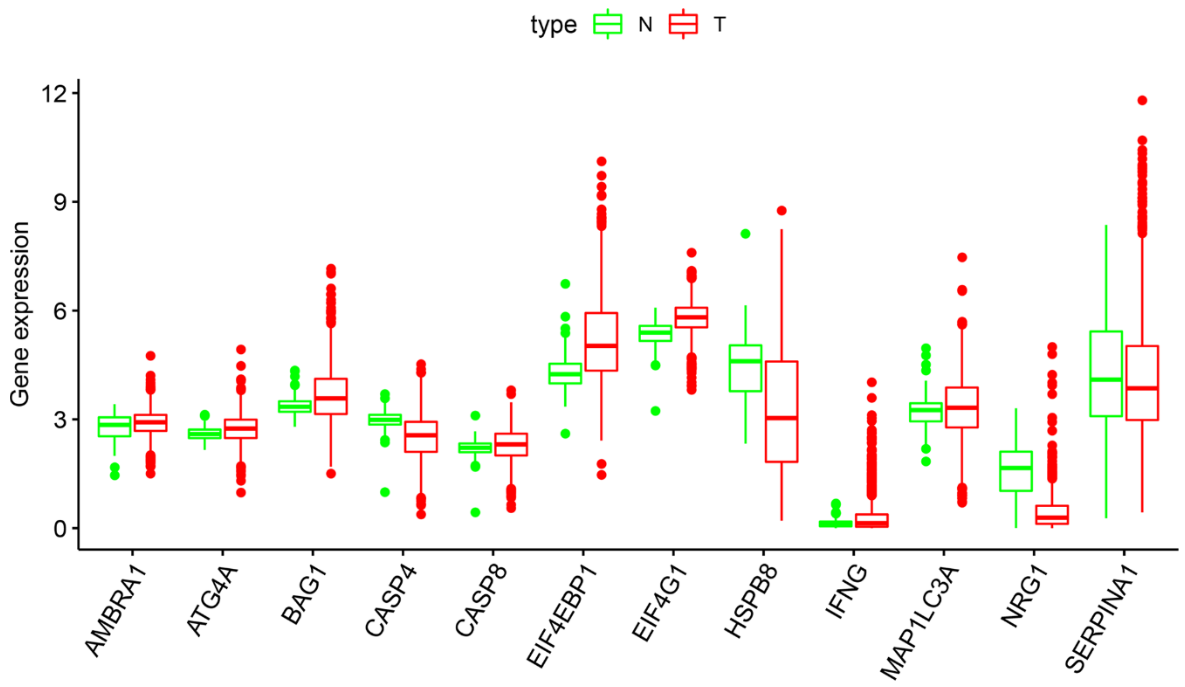

Supplementary Figure 2. The expression patterns of these 12-ARGs.

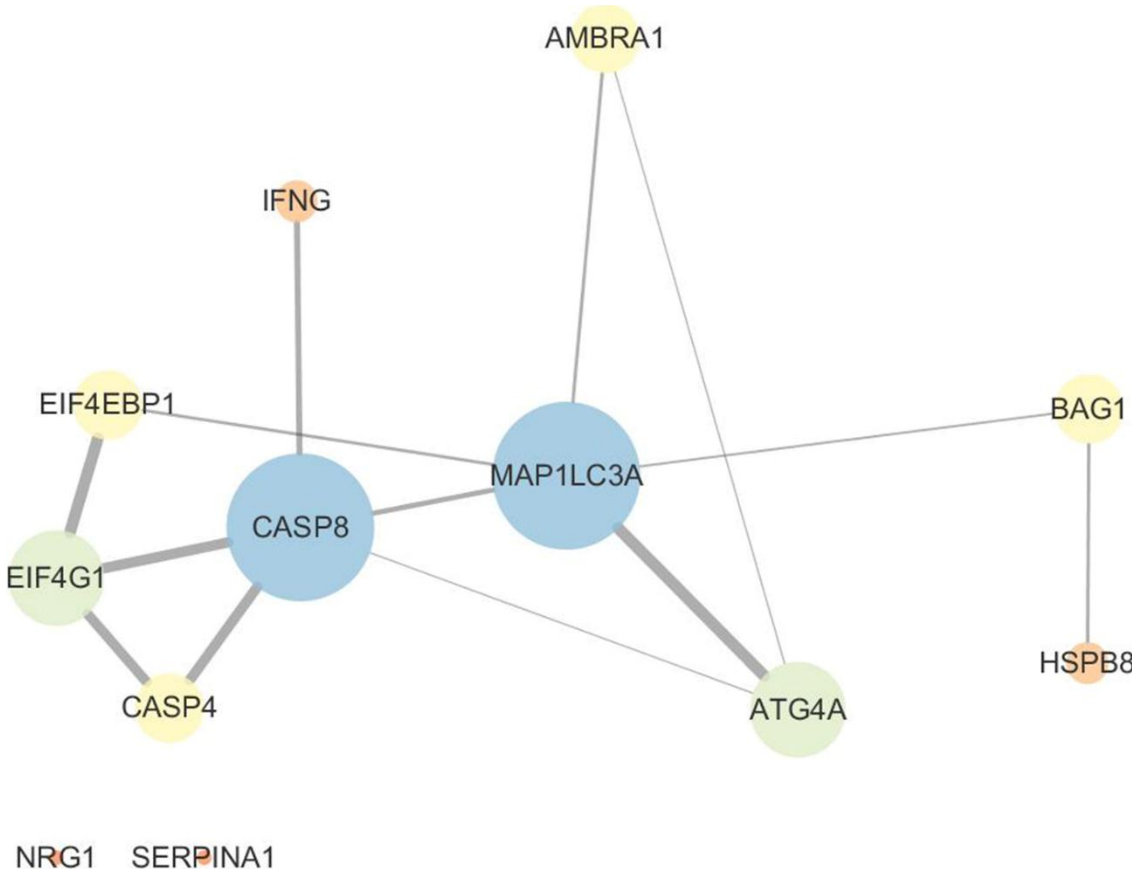

Supplementary Figure 3. Protein-protein interaction (PPI) network of 12-ARGs.
